# Experimental Determination of Residual Stresses Generated by Single Point Incremental Forming of AlSi10Mg Sheets Produced Using SLM Additive Manufacturing Process

**DOI:** 10.3390/ma11122542

**Published:** 2018-12-13

**Authors:** Cecilio López, Alex Elías-Zúñiga, Isaac Jiménez, Oscar Martínez-Romero, Héctor R. Siller, José M. Diabb

**Affiliations:** 1 Honeywell Aerospace, Parque Industrial Ávalos, Vialidad Tabalaopa 8507, Chihuahua 31074, Chih., Mexico; Cecilio.Lopez@honeywell.com; 2 Tecnologico de Monterrey, Escuela de Ingeniería y Ciencias, Ave. Eugenio Garza Sada 2501, Monterrey 64849, NL, Mexico; isaachjc@yahoo.com (I.J.); oscar.martinez@itesm.mx (O.M.-R.); jdiabbzv@itesm.mx (J.M.D.); 3 Department of Engineering Technology, University of North Texas, 3940 North Elm Street, Denton, TX 76207, USA; hector.siller@unt.edu

**Keywords:** additive manufacturing, single point incremental sheet forming, residual stresses, X-ray diffraction

## Abstract

This paper focuses on investigating the residual stress values associated with a part fabricated by Selective Laser Melting technology (SLM) when this is subjected further to forces on single point incremental forming (SPIF) operation of variable wall angle. The residual stresses induced by the SLM manufacturing process on the fabricated AlSi10Mg metallic sheets, as well as those produced during their forming SPIF operation were determined by X-ray diffraction (XRD) measurements. Significant residual stress levels of variation, positive or negative, along the metallic sample were observed because of the bending effects induced by the SPIF processes. It is also shown how the wall thickness varies along the additive manufactured SPIFed part as well as the morphology of the melting pools as a function of the deformation depth.

## 1. Introduction

Additive Manufacturing (AM) is a process of joining materials to produce components from 3D computer data model that are fabricated layer by layer [1]. The purpose of this technology is to improve or replace manufacturing processes commonly used in rapid prototyping since manufacturers have found that the fabrication of models and prototypes rely on complex processes that hindered the development and design phases of new components. The additive manufacturing technology, used in this research called Selective Laser Melting (SLM), was invented by the Fraunhofer Institute Laser Technology in the mid. 1990s. SLM is one of the fastest growing AM technologies worldwide [2,3,4]. This technology has allowed the fabrication of complex components that cannot be made by conventional manufacturing processes. Recent advances show that this technology has expanded its application to metallic-based matrix composites such as the titanium-based materials [5] with improved physical properties and significant cost, production time, and weight reduction when compared to traditional production processes [6]. However, there are still some important issues to be addressed such as fatigue properties, porosity, geometrical accuracy, corrosion properties, wear performance, surface finishing, resulting residual stresses, among others, before the component or system produced by SLM can satisfy certification requirements, standards, international regulations, product life cycling, and intellectual property, among others [7]. Therefore, extensive studies are required to evaluate the mechanical performance of the fabricated SLM parts before these are assembled into a final product. One of these focuses on determining residual stress that are created when a part is processed by SLM technology because of the high temperature gradients, thermal expansion, and non-uniform plastic deformation during heating and cooling cycle.

In fact, residual stresses in metals are created from inhomogeneous plastic deformations because of chemical, thermal, and mechanical fabrication methods. The mechanisms for the developing of residual stresses in AM technologies are due to the temperature gradient since the material strength is instantaneously reduced along the laser beam scan layer because of the heat induced, and to the molten top layers cool-down phase [8,9]. Therefore, temperature gradients that induce non-uniform thermal expansions and contractions surrounding the affected zone will result in additional stresses, while the latter mechanism tends to shrink the underlying material because of its thermal contraction. A review of the major methods for measuring residual stresses is summarized by Huang et al. [10], while numerical techniques to predict residual stresses for selective laser melting process are discussed in [11,12,13,14].

On the other hand, and in order to reduce the undesirable residual stress effects that influence the part mechanical performance, post-processing operations such as shot-peening, grinding, heat treatment, age hardening, or polishing are used to treat the parts fabricated by SLM [15]. These tend to improve the material ductility, increases the fracture toughness threshold value for crack initiation, and reduce surface roughness that affect fatigue performance [16].

Here, in this article, we investigated how residual stresses of a part fabricated by SLM vary when this is subjected further to a manufacturing post-processing operation called single point incremental forming (SPIF). In general, it is not expected that a part fabricated by AM technologies will be subjected to a forming post-processing to reach its final form, however it is of special interest for industrial applications to understand its structural response when this is subjected to external loads that could affect its residual stress magnitudes induced during SLM additive manufacturing process. In this sense, and in order to quantify residual stress distribution induced by the SLM manufacturing process, an experimental technique based on X-ray diffraction (XRD) measurements is used [17,18]. Then, XRD measurements were carried out on the metallic samples to determine residual stresses on the surfaces of the truncated pyramid shape formed by SPIF. Furthermore, we investigated potential effects such as different residual stress levels of variation, positive or negative, along the sample surfaces that were observed during experimental measurements because of the bending stresses induced by the SPIF processes.

## 2. Materials and Methods 

The fabrication of the aluminum samples was carried out as follows: AM technology based on SLM 280^®^2.0 with a maximum working volume of 280 × 280 × 365 mm³ was used to produce a 155 mm × 155 mm aluminum sheet with an average thickness value of 1.2 mm using as based material spherical grain shapes of aluminum metal powder (typically particle size distribution between 20–63 µm). AlSi10Mg powder alloy was used in an argon 5.0 atmosphere to manufacture metallic sheets by considering 200 °C of constant temperature of the building platform. The SLM equipment as well as the powder alloy were supplied by SLM solutions GmbH (Lübeck, Germany). The powder consisted of spherical particles with a size distribution according to the following *D*-values as measured by laser diffraction (Beckman Coulter LS 13320 PIDS, Lübeck, Germany): *D*10 = 30 µm, *D*50 = 43 µm, and *D*90 = 55 µm.

**Figure 1:**
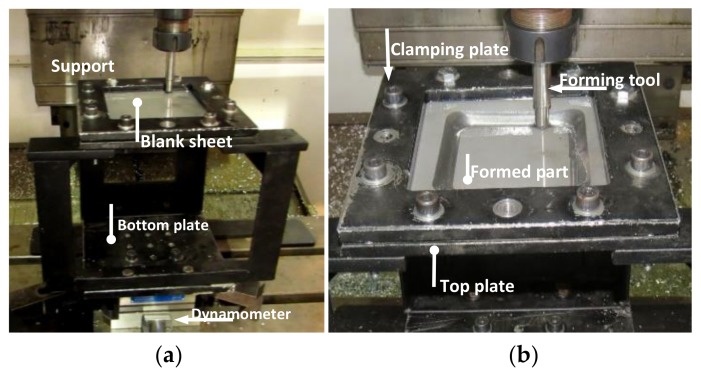
Single point incremental forming experimental setup mounted on a Kryle 9257 CNC milling machine: (**a**) SPIF fixture setup, and (**b**) forming tool with a hemi-spherical tip.

The SLM process involved a layer thickness of 30–50 µm of base material spread uniformly across a metal platform. Then, a laser beam started to scan across it using high-speed XY galvanometers based on the computer-generated programmed path. The focused laser beam melted the powder and fused it to the layer below, after which the platform descended one layer thickness, and the whole process was repeated until the part was finished. The build platform was located inside a metal chamber and was protected with an argon gas to avoid melt pool oxidation [1]. Once the AlSi10Mg sheets were made by SLM, each of these metal samples were clamped into a fixture system of a Kryle CNC 535 (Monterrey, México) three-axis milling machine to form a truncated pyramid part by considering an effective working sample area of 120 × 120 mm^2^, as shown in Figure 1. During the forming process of the aluminum samples, the following SPIF parameters were considered: incremental step Δ*z* = 0.5 mm, feed rate of 3000 mm/min, tool free rotation, initial square length path of 100 mm, varying wall angle, α, starting from 45° until samples failure, a forming tool made of steel with a 10 mm hemi-spherical tip, and mineral oil based lubricant to reduce the friction between the tool and the AlSi10Mg sheet during its forming process.

**Figure 2:**
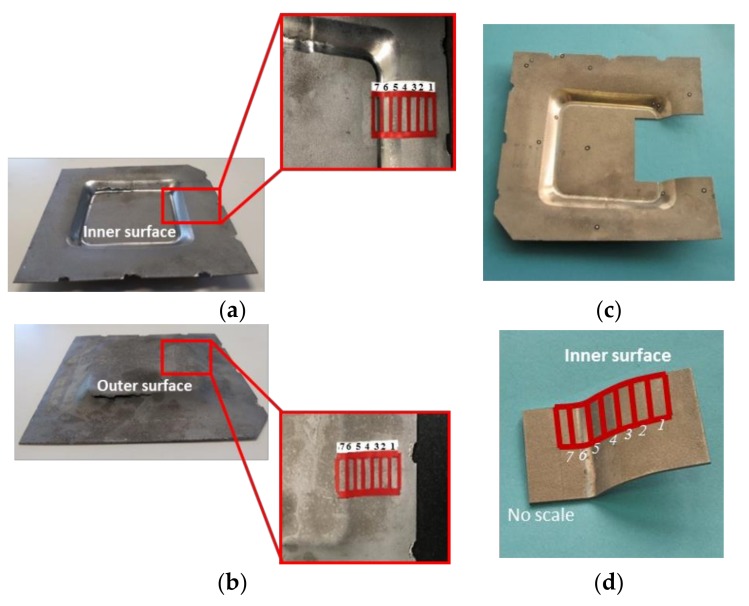
Scanned surface area to determine the residual stress values on (**a**) the inner surface, (**b**) the outer surface, (**c**) cut SPIFed formed part, and (**d**) sample piece used to measure residual stresses.

Once the aluminum sheet samples were formed, a portion of the pyramidal frustum surface were divided into 7 sections to measure the inner and outer surface residual stresses by using X-ray diffraction (XRD) system (see Figure 2). To measure the residual stress on the formed part, the sample was cut by using wire-cut electrical discharge Fanuc Robocut α-C600Ia high performance machine (Chihuahua, México), as illustrated in Figure 2c. Then, a portion of the part, shown in Figure 2d, was mounted in a Panalytical XRD system to measure residual stresses. This experimental process will be discussed next.

## 3. Experimental Measurements

### 3.1. Determination of Residual Stresses

**Figure 3:**
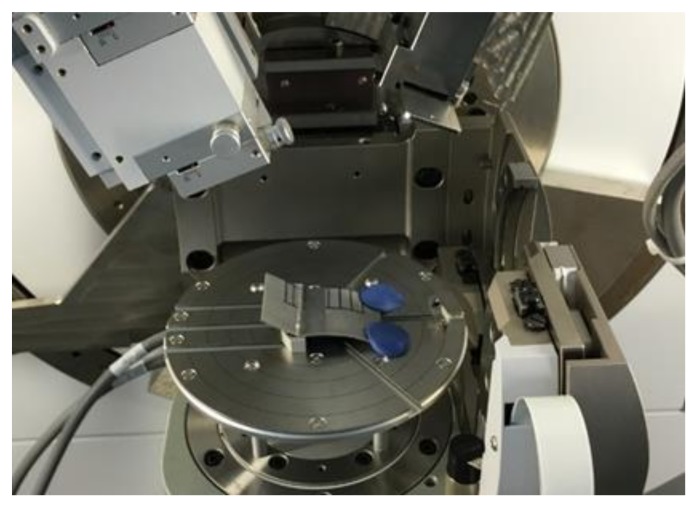
Illustration of the sample regions where residual stress were measured.

**Figure 4:**
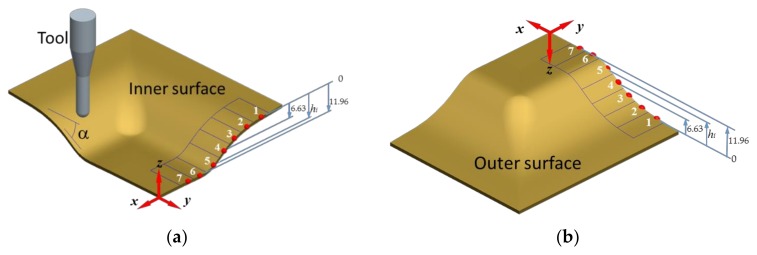
Points on the sample surface at which residual stresses were estimated by XRD technique for (**a**) the inner surface, and (**b**) the outer surface.

A Panalytical Empyrean X-ray diffractometer with α-Cu radiation, at 45 kV and 20 mA, and k-β nickel filter was used to measure residual stresses along the inner and outer surfaces of the formed aluminum samples, as illustrated in Figure 3. The methodology discussed in [17] was followed to estimate the residual stress values along the part forming depth, h_i_, and wall angle α. The residual stress values were computed on the inner and outer sample sections shown in Figure 4a,b by assuming a Poisson ratio value of ν = 0.33, and Young modulus of *E* = 69.5 GPa [19].

**Figure 5:**
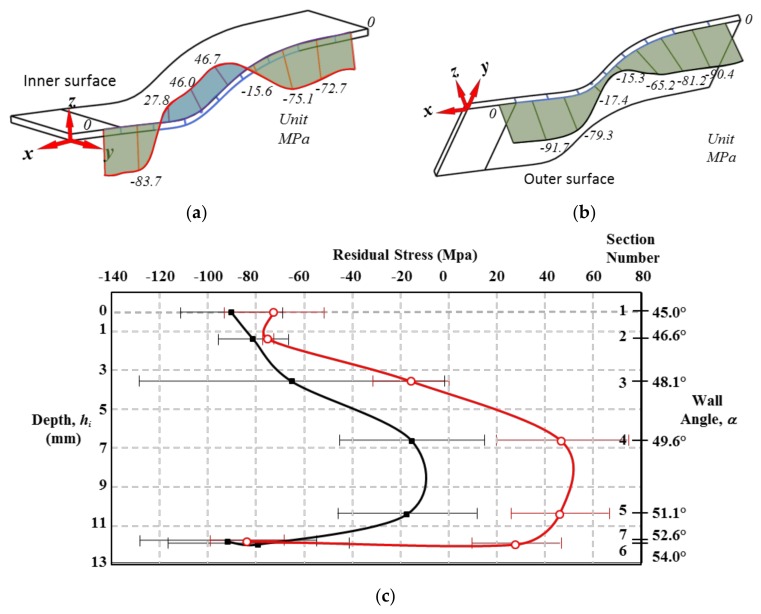
Measured residual stress values on (**a**) the inner surface, (**b**) the outer surface, and (**c**) schematic representation of the residual stress value curves and their standard deviation error bars attained during the XRD measurements as a function of the SPIF process forming depth, *h_i_*, and the wall angle α parameter values.

As shown in Figure 5, the inner and outer forming surfaces are subjected to residual stresses whose magnitude vary as a function of the forming depth and wall angle values. Their maximum collected residual stress values occur at the sections at which the aluminum sheet does not experience any deformation during SPIF process (Sections 1 and 7). Along the plastically formed regions, the inner surface exhibits residual stresses that vary from compressive to tensile values, which is a clear indication of bending effects [17], as illustrated in Figure 5. Table 1 summarizes the recorded residual stresses along the inner and outer surfaces, and the thickness variation in each of the selected sections. Notice from Table 1 that the maximum compressive residual stresses on the inclined surfaces of the truncated pyramid have the magnitude of −91.7 MPa for the outer surface, and of −83.7 MPa for the inner surface. These stress magnitudes were experimentally recorded from the fabricated SLM part before it was post-processed, at the beginning of the sample forming depth. It is observed from Table 1 that for all forming depths of the AM aluminum samples, the outer surface experiences only compressive residual stresses. This is not the case in the inner surface since it has a maximum tensile residual stress of 46.7 MPa recorded at *h_i_* = 6.63 mm, as illustrated in Figure 5. Figure 5 also illustrates the typical standard deviation error bars attained during the XRD measurements whose magnitude values are listed in Table 1. The common sources of these error values are mostly due to the X-ray elastic constants, the apparatus focusing geometry, diffracted peak location, grain size, microstructure, and surface condition. Additionally, preliminary XRD measurements were carried out on the metallic samples in order to determine the initial residual stresses induced by the SLM manufacturing process on both surfaces of the AlSi10Mg metallic sheets and found only compressive stresses with a maximum average value of −92 MPa. As discussed above, this residual stresses change from tensile to compressive ones along the surfaces of the metallic samples when subjected to the SPIF post-processing because of bending effects.

### 3.2. SPIFed Parts Mechanical Modelling

It is well-known that during SPIF process of parts, stretching and bending effects occur. The corresponding equations that describe these phenomena are discussed in [17]. For convenience, we briefly recall some essential relations that describe the mechanical response of parts when subjected to SPIF process.

First, let us consider the elongation strain at which the material samples are subjected during the SPIF process. In this case, thickness strain, better known as the stretching strain at the middle section of the cross sectional area is described by the expression:

(1) $$\varepsilon_{\mathrm{stretching}}=\ln\frac{t_{0}}{t}$$

here *t*_0_ represents the original sheet thickness before deformation, and *t* is the current sheet thickness that varies as a function of the forming depth and the wall angle. For bending effects on SPIFed parts, Malhotra et al. [20] and Jiménez and co-workers [17] concluded that these effects are not negligible during the mechanical response of forming parts since these are responsible for cracks appearance near the maximum sample forming depth. Therefore, bending effects must be estimated if one wants to avoid crack initiation during SPIF process. In this case, bending strain deformation is described by:

(2) $$\varepsilon_{\mathrm{bending}}=\ln\frac{r}{\rho }$$

where r=ρ+y, *ρ* is the theoretical radius of the curvature of the formed part, and *y* is the distance from the middle surface to the element under consideration.

**Figure 6:**
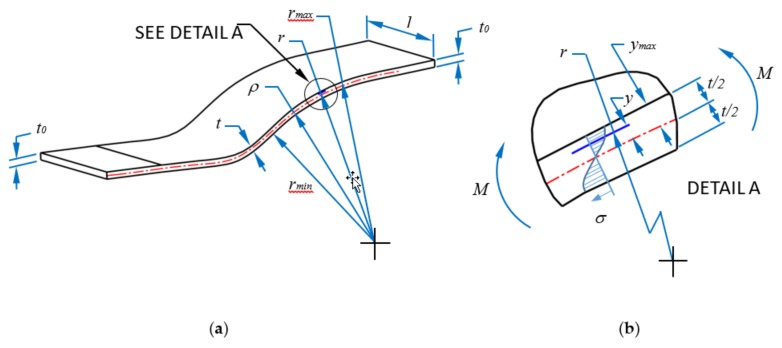

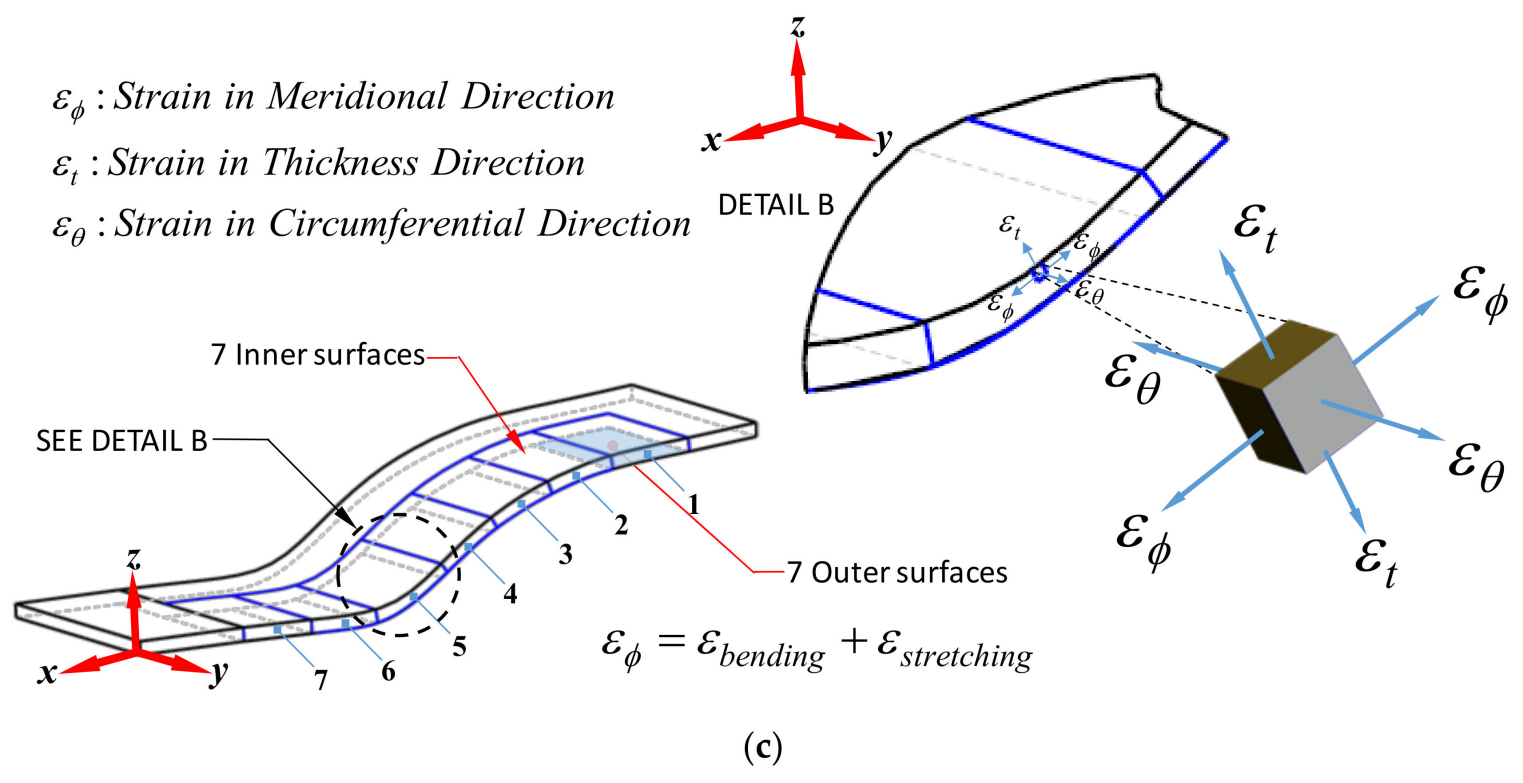
(**a**) and (**b**) Schematic of bending effects acting on the formed part during SPIF, and (**c**) strain deformations experienced by the metallic sheet.

The bending moment, *M*, at which the material is subjected during SPIF process, as a function of the wall thickness sample, can be computed from:

(3) $$M=2K{\left(\frac{1}{\rho }\right)}^{n}\overset{t/2}{\underset{0}{\int }}y^{1+n}dy=K{\left(\frac{1}{\rho }\right)}^{n}\frac{t^{n+2}}{\left(n+2\right)2^{n+1}}$$

where *K* represents the material strength coefficient that for AlSi10Mg has the value of 470 MPa with *n* equal to 0.14. See References [17,21]. Finally, the true stress value that the metallic sheet experiences during SPIF process is computed from the Hollomon expression [21]:

(4) $$\sigma =K\varepsilon^{n}\approx K{\left(\frac{y}{\rho }\right)}^{n}$$

where *y* represents the distance from the middle section to the element under consideration, as shown in Figure 6.

### 3.3. Mechanical Behavior of SLM AlSi10Mg SPIFed Samples

**Figure 7:**
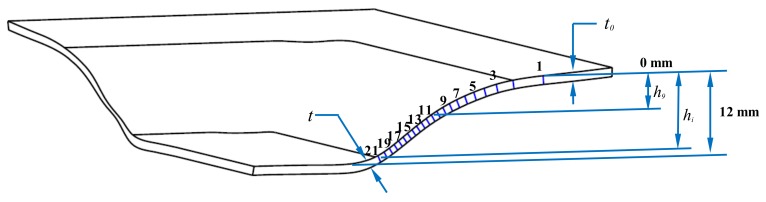
Frontal view of the aluminum sample. Thickness variation along the sample wall was obtained by 3D scan blue light technology equipment.

**Figure 8:**
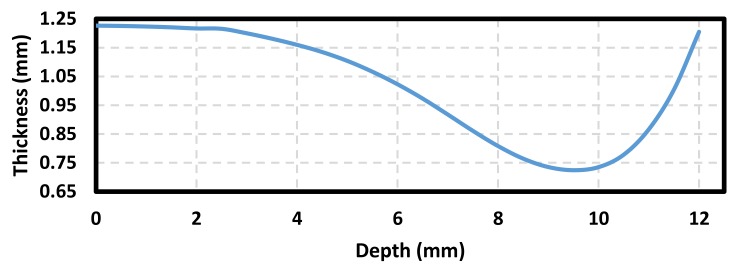
Sample wall thickness variation.

The sample wall thickness was measured from the formed part at the points indicated in Figure 7 by using a 3D scan blue light technology equipment ATOS ScanBox 5108. The collected data is summarized in Table 2. Here, a mean value of 1.226 mm of sheet wall thickness was measured before the AM sheet was SPIFed. Figure 8 illustrates the recorded sample wall thickness variation as a function of the sheet forming depth. These collected values were used to quantify stretching and bending effects of the AM sample through Equations (1) to (4).

**Figure 9:**
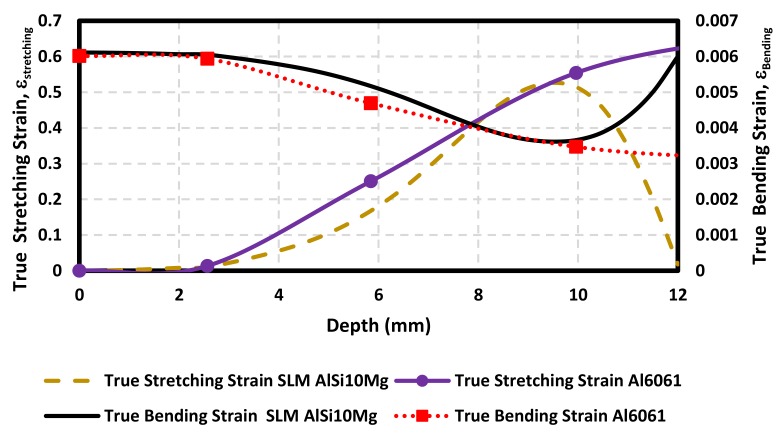
Calculated true stretching and bending strain curves for AlSi10Mg and Al6061.

The samples stretching and bending strains were estimated by considering the variation of the sample wall thickness along the formed part. As one can see from Figure 9, the AM sample stretching strain is higher than bending strains. The maximum value of the stretching strain (0.52 mm/mm) is reached at the forming sample depth of 9.5 mm. At this point, the material sample has been stretched close to its forming limit, and then necking formation takes place, as illustrated in Figure 7 (points 17–21). This is consistent with Young and Jeswiet findings in [22] since the accumulation of high plastic deformation in this region can result in sample damage initiation. In contrast, the maximum value of bending strain (0.00611 mm/mm) occurs at the beginning of the SPIF process. Notice that its value gradually decreases until it reaches a minimum value, at the region at which the stretching strain has a maximum value.

**Figure 10:**
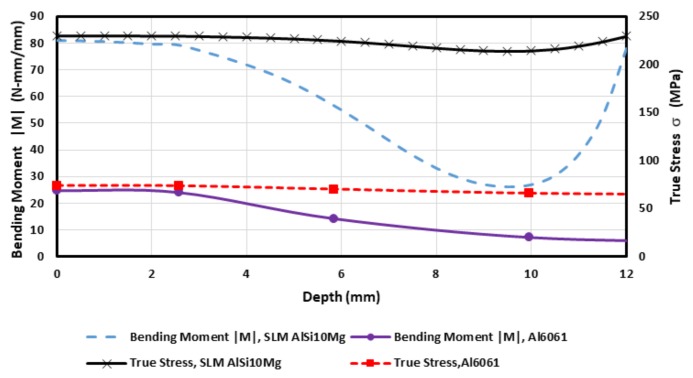
Calculated true stress and bending moment curves.

To have insightful information about the structural behavior of AM aluminum sheets after post processing, bending moment *M* per unit width, and true stresses were calculated by using Equations (3) and (4), respectively. Figure 10 shows the predicted value curves. Notice that both curves tend to reach their maximum value at the beginning of the forming SPIF process. At this stage, the maximum true stress magnitude value was found to be 230.23 MPa, which is close to the material yield stress value of 275 MPa. It is important to mention that the maximum average forming depth value achieved during the samples SPIF process was 12 mm. For exceeding depth values, the metallic samples tend to fracture since the AM process induces, by itself on the metallic sheets, microstructural defects such as pores and overlapping melting pools that hinders their formability limits. In fact, Figure 2b confirms the occurrence of sample fracture close to this region due to meridional stresses [17,20,23].

### 3.4. Comparison of the Mechanical Behavior of SLM AlSi10Mg and Al6061 SPIFed Samples

This section focuses on addressing the principal findings of the mechanical properties of cast aluminum alloys 6061 and SLM AlSi10Mg samples when subjected to the SPIF process. While SPIFed metallic sheets are from different aluminum batches, their mechanical response under large deformations could be of interest for several engineering applications. Therefore, it is believe that this comparison will provide insightful information about the mechanical behavior of Al6061 with respect to post-processed AM parts.

Measurements of the stretching strains of SLM AlSi10Mg and Al6061 samples exhibit slightly different strain behavior mainly due to the work hardening properties of each material. As a result, Al6061 (*n* = 0.2, *K* = 205 MPa) has better stretch-ability at low forming depths than the SLM AlSi10Mg (*n* = 0.14, *K* = 470 MPa), but soon becomes almost of the same magnitude than the AM aluminum samples for forming depth values close to 10 mm, as observed in Figure 9. Furthermore, both material samples exhibit almost the same bending strain behavior, mainly due to the recorded cross-sectional wall thickness of the AlSi10Mg and Al6061 samples used to compute these curves, as illustrated in Figure 10.

**Figure 11:**
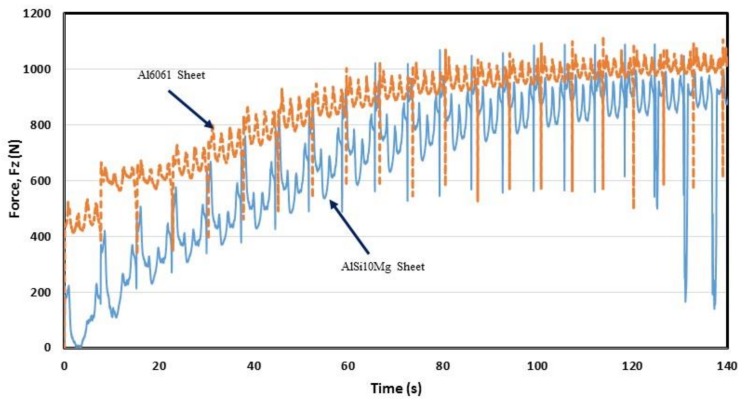
Collected forming forces, *F*_z_, on AM AlSi10Mg and Al6061 SPIFed parts.

The acting forces on SLM AlSi10Mg and Al6061 samples during the SPIfed process were recorded by using a Kistler piezoelectric dynamometer connected to a data acquisition system. Surprisingly, the measured forming forces on the Al6061 samples are higher than those collected on SLM AlSi10Mg sheets, as illustrated in Figure 11. There are several causes that could explain this situation. One is related to the formation of dislocations during the forming process of the material samples. In an ideal situation in which there is no porosity, it would be expected that the cell boundaries would limit dislocation movement during deformation. This increases material strength since the Al grains contain Si particles surrounded by eutectic region, which is indicative of strong interfacial bonding that hinders dislocation motion. See [24,25,26]. Porosity in SLM AlSi10Mg samples are the preferential sites for inhomogeneous deformation and consequently for crack initiation that allows deformation at much lower forming forces, as illustrated in Figure 11.

On the other hand, experimental measurements of residual stresses in the inner and outer surfaces of the SLM AlSi10Mg part, recorded after its SPIFed process, proved to have residual stresses with magnitudes 3 times higher than that recorded in Al6061 samples for the forming depth of 12.6 mm [17]. In fact, SML samples fracture by meridional tensile stresses when the forming depth is about 12 mm, while those of Al6061 failed by circumferential fracture near the forming depth value of 52 mm. Both sample fractures took place at the region of critical thickness reduction [23], i.e., at point 20 of Table 2 for SML AlSi10Mg, and between points 13 and 15 of Table 3. Of course, for additive manufactured parts, fatigue cracks could initiate because of some manufactured defects such as pores and overlapped melting pool regions, near the surface or within the sample interior. This AM sample defects will be addressed in the next section.

## 4. Microstructural Evolution During SPIF Process

**Figure 12:**
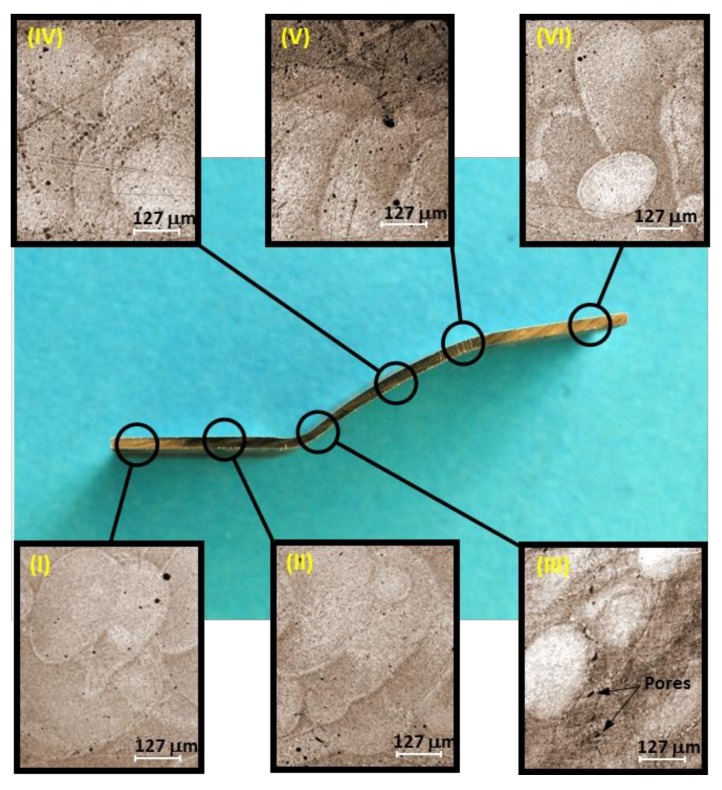
Microstructural evolution of the SPIFed AlSi10Mg part.

In order to study the evolution of the microstructure of AM metallic sheets and the impact that the SPIF forming process has on the melt-pool morphology, SEM images were obtained along the cross sectional area of the formed aluminum samples. Figure 12 shows the optical micrographs evolution along the formed AlSi10Mg etched sample. Six regions have been selected to experimentally obtain, via SEM images, the sample microstructure along its cross-sectional area to understand how the forming process influences the morphology of the melted pools. Elongated melted pools, as well as some pores, are observed from the micrographs illustrated in Figure 12. It is well-known that pores are critical defects since these could initiate inhomogeneous deformation as well as cracks initiation [19,27]. The insufficient overlap observed in Figure 12 of melt pools is detrimental to fatigue life due to the stress concentration at the sharp edges of the pores. Thus, the existence of pores in additive manufactured parts must be controlled by adjusting SLM parameters [28,29] or by subjecting the fabricated part to a post-processing operation. Region IV shows melted pools that have an interwoven form that is mainly due to the hatch spacing between scans in one layer. It is also observed from Figure 12 that the melted pool dimensions vary along the microstructure.

As the forming sample depth increases, the melted pools of Regions V and VI exhibit different morphology. Note that in Region VI, the melt-pool boundaries are well defined. It is noteworthy that in Regions I, II, and III, bending effects take place, mainly due to the contact forces between the aluminum sheet and the forming tool [17,20,30]. Furthermore, the melt-pools change inclination towards the building scanning direction. In these regions, the outer surfaces experienced tensile residual stresses that cause melt-pool and pores elongations. In Region III, pore alignment is evident along the melt-pool building directions. Elongated pore-form is relevant to the sample mechanical properties because of the potential increase in stress concentration magnitude values that could promote crack initiation and propagation that may lead to part premature failure [19,31,32]. 

On the other hand, Figure 2b and Figure 12 exhibits the region of the circumferential failure mode of the truncated pyramid part located close to Region III. In this Region III, the sample experience tensile residual stress of about 27.8 MPa, and reaches its maximum formability capacity since the sample cross-section has the lowest thickness value, as illustrated in Figure 10. Therefore, it is concluded that AM parameters such as scanning direction that influences fatigue life because of the alloy microstructure, smaller hatching spacing that can eliminate pores, power, velocity, and layer thickness that are directly link to melt-pool aspect ratio (length/width) must be appropriately adjusted to prevent premature sample failure during its service life [33,34].

## 5. Conclusions

XDR measurements showed that AlSi10Mg metallic sheets produced by AM technology have only compressive residual stresses on the top and bottom sheet surfaces with a maximum average magnitude value of −92 MPa. However, when these metallic sheets are subjected to SPIF post-process, the magnitude of these stresses change from compressive to tensile values on the inner surface because of bending effects that could initiate part fracture due to meridional stresses. We have also found that the maximum value of the stretching strain occurs for the forming sample depth of 9.5 mm close to the necking formation region for which the material reaches its forming limit. Moreover and during SPIF post-processing of the metallic samples, it was found that after 12 mm of sample forming depth, small cracks appeared on the surface specimens mainly due to AM microstructural defects such as pores and overlapping melting pools that hinders their formability limits. Some defects were observed close to the meridional failure region of the truncated pyramid. At this forming depth, the maximum true stress magnitude value is about 230.23 MPa, which is closed to the material yield stress value of 275 MPa. The experimental samples had sufficient strength to withstand the forming forces that were acting upon them during the SPIF process without failure until they reached a forming depth average value close to 12 mm. However, if sample defects are removed by adjusting the AM parameters and by subjecting the printed metallic parts to the hipping process (hot isostatic pressing for additive manufacturing densification), an increase in the forming depth and fatigue strength will be possible. Therefore, it is concluded that if a part built by metal AM technologies is to be used, a careful examination of its mechanical properties needs to be done before moving it into certification process and full product service. In this sense, this study provides information regarding the variation of residual stresses on the inner and outer surfaces of the metallic samples, and how their magnitude values vary during the SPIF post processing of the fabricated parts in an attempt to emulate their structural response if these are further subjected to external loads during their service life.

## Figures and Tables

**Table 1:** Residual stress values recorded in the inner and outer surfaces of the AM Aluminum sheets as a function of the forming depth.

Section Number	Depth *h_i_* (mm)	Wall Thickness *t* (mm)	Outer Surface		Inner Surface	
			Residual Stress (MPa)	Standard Deviation (MPa) (+/−)	Residual Stress (MPa)	Standard Deviation (MPa) (+/−)
1	0	1.226	−90.4	21.1	−72.7	40.7
2	1.39	1.220	−81.2	14.4	−75.1	4.7
3	3.57	1.060	−65.2	63.6	−15.6	31.5
4	6.63	1.017	−15.3	30.0	46.7	54.4
5	10.41	0.730	−17.4	28.8	46.0	40.5
6	11.96	1.012	−79.3	37.3	27.8	36.4
7	11.81	1.225	−91.7	36.6	−83.7	30.8

**Table 2:** Experimental data that shows wall thickness variation as a function of the sample forming depth.

Point	Depth *h*_i_ (mm)	Thickness *t* (mm)	Point	Depth *h*_i_ (mm)	Thickness *t* (mm)	Point	Depth *h_i_* (mm)	Thickness *t* (mm)
1	0	1.226	8	3.5	1.181	15	7.0	0.917
2	0.5	1.225	9	4.0	1.159	16	7.5	0.860
3	1.0	1.223	10	4.5	1.134	17	8.0	0.808
4	1.5	1.220	11	5..0	1.104	18	8.5	0.764
5	2.0	1.216	12	5.5	1.064	19	9.0	0.735
6	2.5	1.215	13	6.0	1.022	20	9.5	0.724
7	3.0	1.198	14	6.5	0.973	21	10.0	0.735

**Table 3:** Experimental data for Al6061 sample thickness variation as a function of the SPIF depth. Adapted by permission from the International Journal of Advanced Manufacturing Technology [17], Copyright 4486470813200, 2018.

Point	Depth *h*_i_ (mm)	Thickness *t* (mm)	Point	Depth *h*_i_ (mm)	Thickness *t* (mm)
1	2.565	1.1981	10	38.213	0.3817
2	5.842	0.9454	11	41.212	0.3594
3	9.957	0.6978	12	45.024	0.3189
4	14.258	0.6204	13	48.553	0.2755
5	18.833	0.5747	14	51.954	0.3303
6	23.714	0.5329	15	54.682	0.6798
7	27.577	0.4918	16	54.818	1.1588
8	31.253	0.4526	17	54.756	1.2102
9	34.562	0.4281	18	54.874	1.2144
